# **Effect of Vitamin C on Salivary Total Antioxidant Capacity in Smokers**

**Published:** 2012

**Authors:** Sedigheh Bakhtiari, Jamileh Bigom Taheri, Mahin Bakhshi, Hamed Mortazavi, Azadeh Shah Hoseini, Elahe Vahid Dastjerdi, Somayyeh Azimi

**Affiliations:** a *Associate Professor of Oral Medicine Department, Dental School, Shahid Beheshti University Of Medical Sciences, Tehran, Iran.*; b *Professor of Oral Medicine Department, Dental School, Shahid Beheshti University of Medical Sciences, Tehran, Iran.*; c *Assistant Professor of Oral Medicine Department, Dental School, Shahid Beheshti University Of Medical Sciences, Tehran, Iran.*; d *Private Dentist. *; e *Associate Professor of Orthodontics Department, Dental School, Shahid Beheshti University Of Medical Sciences, Tehran, Iran. *

**Keywords:** Total antioxidant capacity, Ascorbic acid, Smoking, Saliva, Abbreviation, Total antioxidant capacity (TAoC)

## Abstract

This study was designed to elucidate the effect of ascorbic acid on salivary total antioxidant capacity in smokers.

In this single blind crossover clinical trial, the whole unstimulated saliva of 30 smokers, who were randomly divided into two groups, was collected. In the first phase after the saliva collection, one group of patients took 500 mg of vitamin C powder, for 3 weeks. Then, saliva of all patients was collected. After a one-week wash-out period, vitamin C was given to the other group. The collection of saliva was done after 3 weeks. Total antioxidant capacity was measured. Statistic evaluation was performed by Repeated Measured ANOVA, Independent sample t-test and Covariate test.

The mean of total antioxidant capacity with and without using vitamin C was 0.511 ± 0.155 (U/mL) and 0.555 ± 0.171 (U/mL), respectively. This variability was not significant (p = 0.605).

Oxidative stress from cigarette smoke was not decreased significantly with using vitamin C.

## Introduction

Smoking increases the incidence of cancer in oral cavity (1). Cigarette smoke is responsible for 50-90% cases of oral cancer (2). The incidence of oral squamous cell carcinoma (OSCC) is 4 to 7 times higher in smokers than non-smokers (3). Cigarette smoke contains over 4,000 chemicals, many types of toxic components, especially free radicals and reactive oxygen (O2-). One “puff” of a cigarette exposes the smokers to over a 1015 free radicals (4). In last few years, increasing evidence has supported the involvement of free oxygen radicals in several human diseases such as cancer (5). It has been suggested that free radicals, reactive oxygen species and reactive nitrogen species in the inhaled cigarette smoke gradually accumulate according to the field cancerization concept, and eventually cause malignant transformation (3). 

Antioxidants protected body against the free radicals (6). Total antioxidant capacity (TAoC) of Saliva consists of enzymatic (*e.g*. superoxide dismutase and peroxidase) and non-enzymatic (*e.g*. uric acid) components (7). 

Cigarette smoke may attack antioxidant system (8). Salivary antioxidants activity in smokers cannot be protective against accumulative stresses. Several studies have demonstrated changes in the activity of salivary antioxidants system in smokers and in patients with SCC comparing with control group (3, 9).

Vitamin C can scavenge free radicals of reactive oxygen (such as super oxide) and reactive nitrogen groups (nitrogen dioxide and) (4).

The idea of using vitamin C for Cancer treatment and Prevention was first suggested in 1949. Investigators demonstrated that administration of high doses of ascorbic acid improves the survival of patients with cancer (10). Recent studies have shown that plasma/serum vitamin C concentrations up to 40% lower in smokers than non-smokers and smokers require higher levels of vitamin C to protect against the oxidative damage (4).

This study was performed to elucidate the effect of ascorbic acid on salivary total antioxidant capacity in smokers.

## Experimental

The present study was designed as a single blind randomized crossover clinical trial. We used the antioxidant assay kit (Cayman chemical, Cat No.709001, and USA) in order to determine the level of TAoC in collected saliva. Patients were selected by simple non-randomized sampling method. Thirty smokers who had been referred to oral medicine department of Shahid Beheshti University of medical sciences (Tehran, Iran) were included in the study. Smokers with the history of 5 or more pack/year of smoking (number of daily cigarette divided to 20, multiply by the number of years with history of cigarette smoking) were included in the study. They had no systemic disease, no history of chemotherapy or radiotherapy, did not take any medications continuously during the past 3 months, did not take alcohol and supplements, and did not have nephrolithiasis. They also had no oral pathology based on clinical examination. Patients were informed about the whole procedure and the consent form was filled up by each patient. Then, they were randomly divided to two groups: A and B.

For the baseline activity, at least 1 mL of each patient’s unstimulated saliva was collected in the beginning of study. Collection of saliva was done in an upright position, between 9 and 12 o’clock in the morning. They had been ordered to avoid eating, drinking, smoking 1 h before the saliva collection. We used 50 mL falcon tubes as saliva containers.

Collected saliva was transferred to laboratory and was placed in specific microtubules immediately. To separate the squamous cells and derbies, samples were centrifuged (5000 g, 4°C) for 15 min (Hetich, Germany). Then, centrifuged samples were preserved at -70°C to be assessed later.

Each groups (A, B) had different instruction for using vitamin C. Patients in group A received 500 mg of solved powder of vitamin C daily (Osveh, Iran) for 3 weeks. In the first phase, patients of group B took no vitamin C and they were asked to follow their routine diet. After 3 weeks, unstimulated saliva samples were collected again.

After a one-week period of washing out, patients of group B were ordered to take vitamin C as mentioned before and cases of group A took no vitamin C. After 3 weeks, unstimulated saliva samples were collected for the third time. These cases, who had forgotten to take vitamin C more than two times in a week, were excluded from the study.

TAoC in collected samples was evaluated using antioxidant assay kit (Cayman chemical, Cat No.709001, USA). The Cayman Chemical Antioxidant Assay Kit measures the total antioxidant capacity of plasma, serum, urine, saliva, or cell lysates. The assay relies on the ability of antioxidants in the sample to inhibit the oxidation of ABTS® (2,2›-azino-di-[3-ethylbenzthiazoline sulphonate]) to ABTS®+ by metmyoglobin. To prevent ABTS oxidation, the capacity of the antioxidants in the sample is compared with that of Trolox, a water-soluble tocopherol analogue, and is quantified as molar Trolox equivalents.

The operator was an experienced technician blind about cases. Each sample was assessed two times to be more accurate. We used ELISA Reader machine (Anthos 2020 Germany) to read the results. Data was statistically analyzed by computer SPSS 15 software. Repeated Measured ANOVA, Independent Sample t-test and Covariate test were used as statistical tests.

## Results

Thirty smoker cases between ages 25 to 70 years (45.23 ± 12.27) participated in this study. The mean of smoking was (16.50 ± 11.32) pack/year. The mean capacity of TAoC in baseline samples was (0.529 ± 0.167) U/mL. The carryover effect was investigated by the independent sample t-test, for comparing two groups, to ensure the sufficiency of washout period and also to make sure that the first phase had not influenced antioxidant capacity in the second phase. Independent sample t-test showed that there was no carryover effect and the washout period was enough (p-value = 0.757). Covariate test confirmed that the age (p = 0.198) and baseline activity (p = 0.969) had no significant effect on the final level of antioxidant. Table 1 demonstrates changes of antioxidant capacity with and without vitamin C under the supervision of statistical specialist. Repeated Measured ANOVA test was used and confirmed that the variability of salivary total antioxidant capacity with and without using vitamin C was not significant (p = 0.605).

**Table 1:** Statistical indices change in enzyme activity in samples

		**Number of samples**	**Minimum**	**Maximum**	**Mean**	**SD**	**Standard error**
**Changes in TAoC**	**Before using Vitamin C**	30	0/257	0/934	0/511	0/155	0/015
	**After using Vitamin C**	30	0/386	0/939	0/555	0/171	0/020

## Discussion

In this study, we used the whole saliva as samples. During the analysis of salivary antioxidants, whole saliva is more relevant as it includes gingival crevicular fluid, immune cells and tissue metabolites (6).

We used powder formulation of vitamin C. Numerous studies have shown that different types of vitamin C have no effect on the rate of its absorption or its efficacy (11).

We have reported earlier that cigarette smoke leads to an elevation in salivary superoxide dismutase activity (12). In addition, in previous study we concluded that vitamin C doesn’t increase the SOD activity in smokers significantly (13). Here, we demonstrate the roll of vitamin C on salivary TAoC. Many studies have demonstrated the total antioxidant status in cigarette smokers (8, 9, 14). Hamo Mahmood *et al. *used cayman chemical antioxidant assay kit in smokers and revealed that there is a significant reduction in total antioxidant status in the smokers (8). In addition, many researchers investigated the effect of vitamin C on saliva in smokers and they had various results (1, 15-17). Panda *et al*. have demonstrated that the oxidative damage of smoking is almost completely prevented by vitamin C (15, 18).

Lee *et al. *used 500 mg of vitamin C to investigate its inhibitory effect on oxidative damage in smokers (1). Numerous studies showed that anticancerous effects of vitamin C are elucidated when used at least 80-110 mg of vitamin C per day (19). We used 500 mg Vitamin C powder. This amount is less than tolerable upper intake level for vitamin C which is 2000 mg/day for adults (20).

In our study, we included smokers with the history of 5 or more pack/year of smoking according to Moller, and Hamo Mahmood (8, 16).

We measured the total antioxidant activity; it is better than measuring the fractional antioxidant. Since the measurement of all known antioxidants is time-consuming, many antioxidants may remain undiscovered and the total activity may be greater than the sum of individual antioxidants due to the cooperative interaction (8).

Our results showed that the use of vitamin C for 3 weeks in smokers does not change TAoC significantly which was in consistent with the result of some studies (21, 22). Klein’s study about investigating the effect of cigarette smoke on oral peroxidase activity in saliva showed that the exposure to cigarette smoke causes a 70% loss of enzyme activity. Many antioxidants’ agents (such as ascorbic acid) were exposed to cigarette smoke and it was demonstrated that they had no protective effect on oral peroxidase (21).

Washio *et al. *showed an increase in plasma level and concentration of vitamin C in hemodialysis patients after using vitamin C supplement. Neither the amount nor the activity of superoxide dismutase had any significant changes in this study (22).

Greabu *et al. *showed that the addition of 10 mg/dL vitamin C to saliva (*in-vitro*) was not able to maintain/restore the salivary antioxidant capacity before smoking. They suggested that an adequate intake of antioxidants such as vitamin C may help smokers to avoid cigarette smoke-induced oxidative damage (2).

## Conclusions

Within the limitation of this study, vitamin C does not increase salivary total antioxidant capacity significantly. Smoke-induced oxidative stresses may be decreased after using more than 3 weeks of vitamin C.

## References

[1] Lee BM, Lee SK, Kim HS (1998). Inhibition of oxidative DNA damage, 8-OHdG, and carbonyl contents in smokers treated with antioxidants (vitamin E, vitamin C, beta-carotene and red ginseng). Cancer Lett.

[2] Greabu M, Battino M, Totan A, Mitrea M M, Totan C, Spinu T, Didilescu A (2007). Effect of gas phase and particulate phase of cigarette smoke on salivary antioxidants. What can be the role of vitamin C and pyridoxine?. Pharmacol. Reports.

[3] Nagler RM, Reznick AZ (2004). Cigarette smoke effects on salivary antioxidants and oral cancer-novel concepts. Isr. Med. Assoc. J.

[4] Northrop-Clewes CA, Thurnham DI (2007). Monitoring micronutrients in cigarette smokers. Clin. Chim. Acta.

[5] Garg N, Singh R, Dixit J, Jain A, Tewari V (2006). Levels of lipid peroxidase and antioxidants in smokers and non-smokers. J. Periodontal Res.

[6] Scully DV, Langley-Evans SC (2002). Salivary antioxidants and periodontal disease status. Proc. Nutr. Soc.

[7] Nagler RM, Klein I, Zarzhevsky N, Drigues N, Reznick AZ (2002). Characterization of the differentiated antioxidant profile of human saliva. Free Radic. Biol. Med.

[8] Hamo Mahmood I, Abdullah KS, Othman SH (2007). The total antioxidant status in cigarette smoking individuals. MJBU.

[9] Nagler RM (2007). Altered salivary profile in heavy smokers and its possible connection to oral cancer. Int J. Biol. Markers.

[10] Li Y, Schellhorn HE (2007). New developments and novel therapeutic perspectives for vitamin C. J. Nutr.

[11] Choi HK, Gao X, Curhan G (2009). Vitamin C intake and the risk of gout in men: a prospective study. Arch. Intern. Med.

[12] Baharvand M, Maghami AG, Azimi S, Bastani H, Ahmadieh A, Taghibakhsh M (2010). Comparison of superoxide dismutase activity in saliva of smokers and non-smokers. South Med. J.

[13] Bakhtiari S, Baharvand M, Anbari F, Azimi S, Taheri JB (2011). Effect of vitamin C on salivary superoxide dismutase activity in smokers. Afr. J. Biotechnol.

[14] Buduneli N, Kardeşler L, Işik H, Willis CS 3rd, Hawkins SI, Kinane DF, Scott DA (2006). Effects of smoking and gingival inflammation on salivary antioxidant capacity. J. Clin. Periodontol.

[15] Panda K, Chattopadhyay R, Chattopadhyay D, Chatterjee IB (2001). Cigarette smoke-induced protein oxidation and proteolysis is exclusively caused by its tar phase: prevention by vitamin C. Toxicol. Lett.

[16] Moller P, Viscovich M, Lykkesfeldt J, Loft S, Jensen A (2004). Vitamin C supplementation decreases oxidative DNA damage in mononuclear blood cells of smokers. Eur J. Nutr.

[17] Schneider M, Diemer K, Engelhart K, Zankl H, Trommer WE, Biesalski (2001). Protective effects of vitamins C and E on the number of micronuclei in lymphocytes in smokers and their role in ascorbate free radical formation in plasma. Free Radic. Res.

[18] Panda K, Chattopadhyay R, Chattopadhyay DJ, Chatterjee IB (2000). Vitamin C prevents cigarette smoke-induced oxidative damage in-vivo. Free Radic. Biol. Med.

[19] Carr AC, Frei B (1999). Toward a new recommended dietary allowance for vitamin C based on antioxidant and health effects in humans. Am. J. Clin. Nutr.

[20] Weinstein M, Babyn P, Zlotkin S (2001). An orange a day keeps the doctor away: scurvy in the year 2000. Pediatrics.

[21] Klein I, Nagler RM, Toffler R, van Der Vliet A, Reznick AZ (2003). Effect of cigarette smoke on oral peroxidase activity in human saliva: role of hydrogen cyanide. Free Radic. Biol. Med.

[22] Washio K, Inagaki M, Tsuji M, Morio Y, Akiyama S, Gotoh H, Gotoh T, Gotoh Y, Oguchi K (2008). Oral vitamin C supplementation in hemodialysis patients and its effect on the plasma level of oxidized ascorbic acid and Cu/Zn superoxide dismutase, an oxidative stress marker. Nephron Clin. Pract.

